# Numerical simulation of air age in dental offices

**DOI:** 10.1038/s41598-022-18588-9

**Published:** 2022-08-19

**Authors:** Eriko Nambu, Kazunori Nozaki, Makoto Tsubokura, Mikako Hayashi

**Affiliations:** 1 Division for Medical Information, Osaka University Dental Hospital, 1-8, Yamadaoka, Suita, 565-0871 Japan; 2 Department of Computational Science, Graduate School of System Informatics, Kobe University and RIKEN Center for Computational Science, Kobe, Japan; 3 Department of Restorative Dentistry and Endodontology, Osaka University Graduate School of Dentistry, Suita, Japan

**Keywords:** Biomedical engineering, Infection control in dentistry

## Abstract

Dental professionals are at high risk of exposure to communicable diseases during clinical practice, but many dental clinics provide clinical care in closed spaces. Therefore, it is essential to develop efficient ventilation methods in dental clinics that do not rely on natural ventilation. In this study, to clarify the factors that cause air retention in dental offices, we conducted computational flow dynamics simulations focusing on (1) the flow path from the entrance to the exhaust port and (2) the presence of partitions. A three-dimensional model of a dental clinic with three dental chairs was created, and simulations were conducted for scenarios with and without partitions with different entrance and exhaust port positions. Evaluation of these simulations on the basis of the age of air, an indicator of ventilation efficiency, showed that the value of the air age near the partition was locally high in the scenarios with partitions. In the scenarios where the exhaust port was located close to the entrance, the air age near the exhaust port was high, regardless of the presence of a partition. In addition to wearing protective clothing and sterilizing instruments, it is important to consider air quality improvement as a countermeasure against airborne and droplet infections, such as virus infections, in dental clinics.

## Introduction

Dental professionals are at high risk of exposure to communicable diseases during clinical practice^1^. Therefore, in dental care, surgeons routinely wear masks and gloves during treatment, and disinfect and sterilize the instruments used to prevent infection within the breathing zone of the surgeon and patient^2^. Nevertheless, with the spread of coronavirus disease 2019 (COVID-19), in addition to the conventional infection control measures based on standard precautions^3^, efforts to further reduce the risk of contracting infections are required in dental care.

Although there is some debate as to whether COVID-19 is transmitted by droplets or is airborne^4–6^, it is a respiratory virus, and airborne transmission is the main mode of transmission of respiratory viruses^7^. As a part of the infection control measures, the WHO specifies standards for natural ventilation in health facilities that handle infections caused by droplets and droplet nuclei^8^. Although some reports have described the evaluation of contaminant dilution by CFD analyses in naturally ventilated dental clinics^9^, dental clinics usually have air conditioners running at all times to ensure the comfort of the surgeon and patients. To maximize the effectiveness of air conditioners, and to avoid the interference caused by sunlight and wind, surgeons and patients spend long hours with the windows and curtains closed, necessitating an efficient ventilation method that does not rely on natural ventilation.

Ventilation is the process of supplying external air to a space or building through natural or mechanical means^10^. Among the main ventilation systems in a dental office, the mechanical agents responsible for ventilation are exhaust vents and air conditioners. Examination room entrances are usually open, allowing inflow of natural air. An efficient ventilation system has been reported to reduce the risk of airborne transmission of COVID-19 indoors^11^, and rapid removal of the virus-contaminated indoor air without recirculating it is important in this regard. The European Research Association for Heating, Ventilation, and Air Conditioning (REHVA) also recommends that heating, ventilation, and air conditioning (HVAC) systems should be used to stop air recirculation and increase the inflow of outside air^12^. In dental clinics that do not have natural ventilation and are not equipped with filters to filter recirculated air or HVAC systems to exhaust indoor air to the outside and bring in fresh air, the contaminated air in the room may recirculate and increase the concentration of contamination.

Since the location of the entrance and exit points has been shown to affect the concentration of indoor air pollutants^13^, appropriate positioning of the exhaust port may be important in dental offices. However, since changes in the positioning of the exhaust vent cannot be easily performed in an existing dental office, it is necessary to examine whether increasing or decreasing the ventilation volume can improve air quality. Other factors that affect air flow in a dental office include partitions. In a typical dental clinic, multiple treatment chairs are arranged in rows at regular intervals in a single treatment room. In many cases, partitions are placed between chairs to provide privacy to patients during treatment. In newly built offices, the attenuation of volatile organic compound (VOC) concentrations in the space, such as those from adhesives, has been shown to depend on the layout of the partitions^14^. Among the equipment installed in a dental office, partitions can be easily rearranged; however, the effect of partitioning on the air quality in a dental office has not been determined.

Therefore, in this study, we focused on (1) the flow path from the inlet to the exhaust port and (2) the presence of partitions as factors that may affect air quality in a typical dental office. Using air age, an index of ventilation efficiency, CFD simulations were conducted to clarify which factors have the most influence on air age and cause air stagnation.

A limitation of this study is that it is a numerical experiment based on simulations and has not been validated by experiment. However, there is currently no experimental method to easily measure the air age of a clinic room. Therefore, simulation is the first choice. SVE3 as a simulation method for evaluating room ventilation efficiency is a method that has already been trusted in various ventilation volume evaluations. Some experiments on the distribution of droplet dispersal sedimentation during dental treatment with fluorescein dye have shown the effectiveness of extraoral vacuums^15^. However, it shows the concentration on the wall surface where the droplets were dispersed, not the concentration of air contamination in the treatment room space. This is different from the evaluation of air quality, which was the purpose of this study. Therefore, the study argues for general ventilation.

## Materials and methods

**Figure 1:**
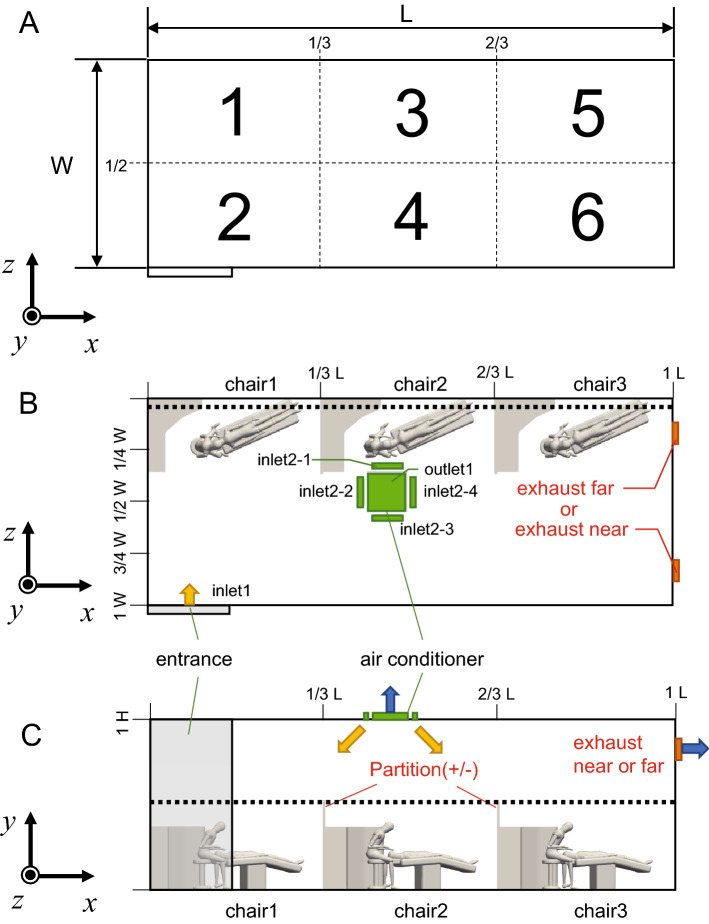
3D geometry of the dental room used in the computational flow dynamics simulation. (**A**) The dental office was divided into six zones when viewed from the y-direction to evaluate the simulation results. (**B**) Arrangement of the geometry in the dental office when viewed from the y-direction. (**C**) Arrangement of the geometry in the dental office when viewed from the z-direction.

In the paper by Zhao et al.^16^ CFD was performed using the geometry of a typical office interior space. In this study, following this approach, CFD analysis was performed using a 3D geometry of a dental office space created with CAD software (Fusion360, Autodesk) based on a 2D drawing of a typical dental office to numerically evaluate air age. The width, height, and depth of the dental office were set to be 9.8 m (*x* direction), 2.9 m (*y* direction), and 4.0 m (*z* direction), respectively. The geometry of the three patient chairs, three dentist chairs, and three dental cabinets was reproduced for analysis (Fig. 1). For each chair, mannequins were used to represent patients and dentists. For the air conditioning system, one open entrance, one exhaust outlet, and one air conditioner were reproduced, as shown in Fig. 1B and C. The chair installed closest to the entrance was designated Chair1; the chair installed closest to the exhaust outlet was designated Chair3; and the chair installed between the two was designated Chair2. In this study, natural ventilation from open windows was not assumed. Using this three-dimensional shape as a reference, the position of the exhaust vent and the presence of partitions was changed depending on the implementation scenario. The partitions were set at a height of 1.7 m (2/3*H*) above the floor level and were in contact with the cabinets of the chairs installed in the center of the room and on the exhaust port side.

OpenFOAMv7, an open-source CFD software, was used for the CFD analysis. SimpleFOAM was selected as the solver, and an incompressible steady flow analysis was performed using a Reynolds-averaged turbulence model. An isothermal field was used, and the heat-transport equation was not solved. The wall conditions were set to adiabatic nonslip conditions. After the start of inflow, 300 s were observed for the calculation to stabilize in the steady-state analysis. The inflow and outflow conditions were set at one inlet/outlet, four air-conditioner outlets, one air-conditioner inlet, and one exhaust outlet, as shown in Fig. 1C. The exhaust outlet was defined as "exhaust-far" when it was located close to the entrance and "exhaust-near" when it was located far from the doorway in Fig. 1B. The details were as shown in the values in Table 1. The air-conditioner was set to the flow-only mode and strongest wind setting, and the boundary conditions were given assuming unfiltered circulating air. The ventilation volume in the examination room was set to less than twice the volume.

**Table 1:** The boundary conditions: Air-conditioner vents, ventilation of the dental office (assuming a capacity of six people), exhaust vents (whose location may change depending on the simulation scenario), entrance.

Air conditioner	Outlet (total of 4 locations)	Airflow (strong wind)	0.29 m^3^/s
	Outlet (per location)	area	0.03 m^3^
		air volume	0.07 m^3^/s
		wind's direction	θ = 45°
		velocity (perpendicular to blowing surface)	x direction 2.42 m/s, y direction − 2.42 m/s
		velocity	3.42 m/s
Clinical room ventilation volume		floor space	39.20 m^2^
		Exclusive area (per person)	6.50 m^2^
		Required ventilation (per person)	30 m^3^/h
		Clinical room ventilation volume	360 m^3^/h
Exhaust (Change the installation position depending on the case)		Surface area	0.09 m^2^
		velocity	1.11 m/s
Entrance		Surface area	4.35 m^2^
		velocity	0.02 m/s
Others		-	Wall, Non-slip, Insulated

Air age was used as an index to evaluate the ventilation efficiency of dental office geometry. The evaluation of indoor air quality by air age has been reported in previous studies in hospital rooms with beds^17,18^ and offices with natural ventilation^19^. It is possible to calculate the air age distribution in a space using SVE3 (scale for ventilation efficiency 3)^20^ proposed by Murakami and Kato^21^ shows ventilation efficiency by CFD using air age. The shorter the time taken for the influent air to reach a given point in the dental office, the smaller the possibility that the air is contaminated, and the longer the time, the greater the possibility of air contamination.

The value of air age for the entire three-dimensional model space of the dental office output as a result of the calculation was nondimensionalized by the local average air age of the exhaust port, and the space was divided into six zones, zone1 ~ 6, as shown in Fig. 1A. The sum of the air ages in each zone was calculated and compared. The sum of the air age for each zone was calculated using Eqs. (1)–(4).

1 $$ cell\left( {\varvec{X}} \right) = \mathop \sum \limits_{k}^{N1} cell.V\left[ k \right] $$

2 $$ age\left( {\varvec{X}} \right) = \mathop \sum \limits_{{i < cell\left( {\varvec{X}} \right)}}^{N1} \left( {\frac{cell.V\left[ i \right]}{{cell\left( {\varvec{X}} \right)}} \times \tau \left[ i \right]} \right) $$

3 $$ exhaust = \mathop \sum \limits_{{i < cell\left( {\varvec{X}} \right)}}^{N1} \left( {\frac{cell.V\left[ i \right]}{{cell\left( {\varvec{X}} \right)}} \times \tau \left[ i \right]} \right) $$

4 $$ age\left( {\varvec{X}} \right) = \frac{{age\left( {\varvec{X}} \right)}}{exhaust} $$

In Eq. (3), where *k* is the volume of each cell and *cell.V*[*k*] is the volume of each cell, the total number of cells in each zone, *cell*(***X***), is calculated. In Eq. (4), for the number of cells in each zone, the volume of each cell, *cell, V* [*i*], is divided by the total number of cells, *cell*(***X***), and the air age of each *cell*, $\tau \left[i\right]$, was summed and added together to calculate the total air age of the zone, *age*(***X***). The average air age of the exhaust outlet was calculated using exhaust and divided by the sum of the air ages to normalize the air age of the zone.

The cross-section used to visualize the calculation results is shown in Fig. 1B and C. To show the effect of partitions, the horizontal cross-section was set to the height of the center of the partition (1/2*H*). To observe the areas where the air is expected to be particularly stagnant, the vertical cross-section was set near the installation surface of the partition and wall (1/9* W*).

Four scenarios were proposed and simulated as shown in Table.2, depending on the location of ventilation openings and partitions. The case with a partition is denoted as (+) and the case without a partition is denoted as (−). The case where the exhaust port is close to the inlet is denoted as "near", and the case where it is far from the inlet is denoted as "far".

**Table 2:** Four scenarios were proposed for simulation depending on the location of the ventilation openings and the presence of partitions.

		Exhaust vent	
		Near	Far
Partition	(+)	SCENARIO A	SCENARIO B
	(−)	SCENARIO C	SCENARIO D

## Results

**Figure 2:**
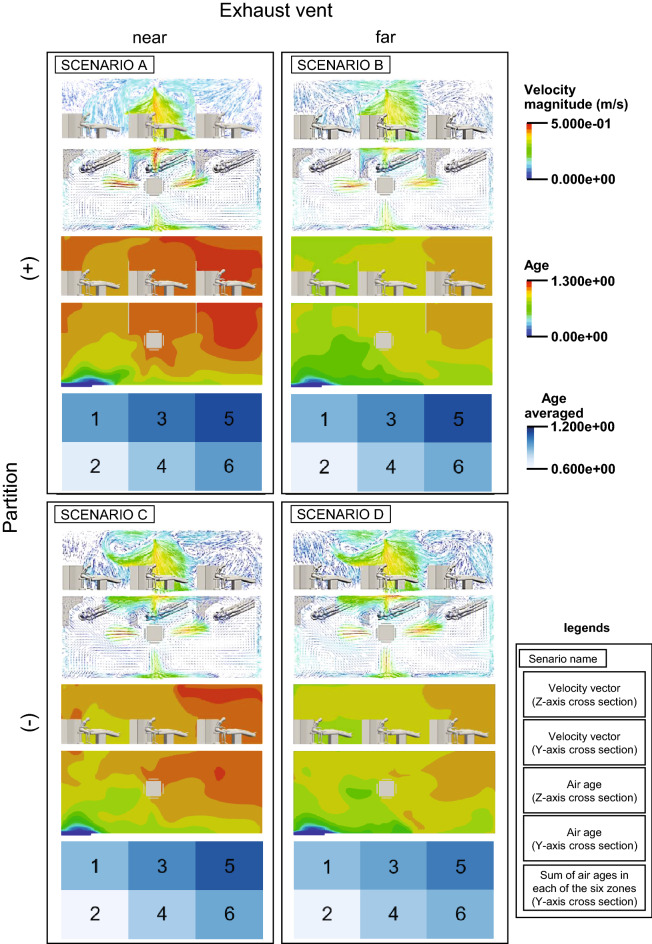
Visualization of the computational flow dynamics simulation results. Velocity magnitude: the velocity vector field of the entire dental office was visualized, Age: visualization of passive scalar values of age of air for the entire dental office, Age averaged: The dental office was divided into six zones, and the average air age in each zone was visualized.

Figure 2 shows the differences in the simulation results depending on the position of the ventilation openings and presence of partitions. The upper two figures show the flow field in the dental office space in the longitudinal and transverse sections. In scenarios C and D, the airflow spreads throughout the room, while in scenarios A and B, the airflow from the air conditioner flows along the partition, indicating that the airflow is affected by the partition. In scenarios B and D, where the exhaust vents are farther away, the circulating flow near the side walls (*W* = 1, *L* > 2/3) is different from those in scenarios A and C, where the vents are closer to each other.

The middle two figures show the air age of the dental office space in the longitudinal and transverse sections, and the air age values near the partitions in scenarios A and B are higher because of the installation of partitions. The air age in Zone 5 is higher than that in Zone 6 in scenarios A-D, particularly in scenarios A and C, where the exhaust vents were close to each other.

The lower figure shows the results of dividing the cross-sectional area of the dental office into six zones and calculating and visualizing the spatial mean values of air age in each zone with and without partitions and the location of the exhaust vents. The spatial mean value of air age in each zone, with or without partitions, did not differ significantly. A comparison of the spatial mean values of air age for different locations of the exhaust vents showed that the values in zones 3, 5, and 6 are especially high in scenarios A and C, where the exhaust vents are close to each other, while the values in scenarios B and D, where the exhaust vents are far from each other, are lower in all zones than in scenarios A and C, where the exhaust vents are close to each other. In particular, in scenarios A and C, the spatial mean value in Zone 5 was higher than that of the other zones regardless of the presence or absence of partitions, indicating that the location of the exhaust vent affects the air age value.

## Discussion

The locations of the inlet and exhaust ports affect the concentration of indoor air pollutants, including in general work rooms^7^ and operating rooms^22,23^. The results of this study showed that, in the exhaust-far scenario, the spatial mean value of air age per zone was generally lower than that in the exhaust-near scenario. The reason for this may be that in the exhaust-near scenario, the flow path of fresh air is short-circuited because of its proximity to the inlet and outlet, resulting in a shorter residence time, and the air is discharged before it is sufficiently mixed with the polluted air in the room. This result was consistent with the trends observed in general workroom spaces^13^. In the scenarios of the dental practice space and the workroom reproduced in this study, the air flows horizontally from the entrance to the exhaust port, but this does not match the trend of this study because in the ventilation of operating rooms, the ventilation direction is generally vertical and multiple exhaust ports may be installed.

In Japan, the "Act on Securing Sanitary Environments in Buildings (Building Management Act)," considers the number of people and the time they stay in relation to the volume of the room as an index of indoor ventilation^24^. In the UK, the Building Regulations 2002^25^, and in the United States, the ASHRAE standard^26^ have been established. Therefore, it is necessary to consider the amount of ventilation required to improve the air quality in dental clinics. The ventilation rate assigned to the simulation in this study was within the maximum allowable value of the index of the Japanese Building Management Law; however, it was clear that sufficient ventilation could not be obtained in the dental office under this condition.

The attenuation of VOC concentrations in a space has been shown to depend on the partition layout^14^. Although the partitions installed in the dental office analyzed in this study affected the airflow, the spatial averages of air age in each zone showed minimal differences. Partitions between the chairs in a real dental office are necessary for patient privacy. However, some localized areas of high air age in the dental office may be overlooked because of averaging. For example, when partitions were installed between the chairs, the air tended to stagnate in the space surrounded by the wall and partitions as in Scenario A in Fig. 2. This trend is consistent with the results of an evaluation of the effect of partitions on the ventilation performance when partitions are installed to block the airflow between the room entrance and exit, as reported in a previous study^27^.

For efficient ventilation in dental offices that do not rely on natural ventilation, it is necessary to consider the location of exhaust vents and the increase or decrease in ventilation volume with partitions in place. In addition to wearing protective clothing and sterilizing instruments^2^, it is important to consider air quality improvement as a countermeasure against airborne and droplet infections, such as virus infections, in dental clinics.

Although standards for infection control of respiratory viruses, which are mainly transmitted through the air, have been established in healthcare facilities^8^, establishment of clear standards for ventilation in accordance with the standard precautions is essential in dental clinics, which are closed for long periods of time without natural ventilation and where air conditioners are constantly operated. Thus, there is a need to establish clear standards for ventilation in accordance with standard precautions^3^.

In the three-dimensional geometrical reproduction of the dental office used in this study, a layout with three patient chairs arranged in the same row was adopted. Although the structural and equipment standards for dental treatment rooms are in accordance with the Ordinance for Enforcement of the Enforcement Regulation on the Medical Care Act^28^, there are no specific rules or standards for setting the locations of examination tables, cabinets, partitions, air conditioners, exhaust vents, and entrances and exits. These standards are expected can expected to provide a safe and secure environment for dental professionals and patients by setting standards for the installation of infection control equipment in dental offices and facilitating improvement of the air environment. These findings will also serve as the foundation for additional studies evaluating measures to ensure appropriate infection control in dental clinics.

## Data Availability

The datasets used and/or analyzed during the current study available from the corresponding author on reasonable request.
